# Local Treatment of Metastatic Prostate Cancer: What is the Evidence So Far?

**DOI:** 10.1155/2018/2654572

**Published:** 2018-03-19

**Authors:** Pedro Leonel Almeida, Bruno Jorge Pereira

**Affiliations:** Faculdade de Ciências da Saúde, Universidade da Beira Interior (FCS-UBI), Covilhã, Portugal

## Abstract

**Background:**

Advances in technological, laboratorial, and imaging studies and new treatments available in the last decades significantly improved prostate cancer survival rates. However, this did not occur in metastatic prostate cancer (mPCa) at diagnosis which, in young and fit patients, will become invariably resistant to the established treatments. Progression will lead to an impairment in patients' quality of life and disease-related death.

**Methods:**

The authors intend to perform a literature review of the advantages of primary treatment of mPCa. Articles were retrieved and filtered for relevance from PubMed, SciELO, and ScienceDirect until March 2017.

**Results:**

Primary treatment is currently indicated only in cases of nonmetastatic PCa. Nonetheless, there might be some benefits in doing local treatment in mPCa in order to control local disease, prevent new metastasis, and improve the efficacy of chemotherapy and hormonotherapy with similar complications rate when compared to locally confined cancer. Independent factors that have a negative influence are age above 70 years, cT4 stage or high-grade disease, PSA ≥ 20 ng/ml, and pelvic lymphadenopathies. The presence of 3 or more of these factors conditions CSS and OS is the same between patients who performed local treatment and those who did not. Metastasis degree and location number can also influence outcome. Meanwhile, patients with visceral metastases have worse results.

**Conclusions:**

There is growing evidence supporting local treatment in cases of metastatic prostate cancer at diagnosis in the context of a multimodal approach. However, it should be kept in mind that most of the existing studies are retrospective and it would be important to make consistent prospective studies with well-defined patient selection criteria in order to sustain the existing data and understand the main indications to select patients and perform primary treatment in mPCa.

## 1. Background

Prostate cancer (PCa) is the fourth most common neoplasm worldwide with about 75% of the diagnoses performed in developed countries. Around the world, 307.000 deaths are estimated due to PCa, thus ranking as the fifth cause of death (6.6%). It is the second most common neoplasm in European men above 70 years and the second cause of death due to oncological disease. 10 to 20% may be metastasized at diagnosis [1–3]. This article intends to review the current literature on primary treatment of metastatic prostate cancer, inferring whether there is an advantage in terms of clinical outcome and trying to define criteria for its application.

## 2. Methods

A vast literature review was carried out using mainly PubMed, SciELO, ScienceDirect, and publications of the International Journal of Urology. During the research, the most relevant articles published were identified, with preference for articles published in the last 10 years, until December 2016.

The research was conducted in the English language, with a free text protocol including preferably the following terms: “metastatic prostate cancer,” “primary treatment,” “local treatment,” “radical prostatectomy,” “radiotherapy,” “cytoreductive surgery,” and “cytoreductive treatment.” The references of the articles used were carefully analyzed to find publications with pertinent information, which eventually were included in the present dissertation.

## 3. Results and Discussion

### 3.1. Primary Treatment of PCa

Currently, primary treatment in PCa is indicated as first line in cases of nonmetastatic PCa and may be performed as palliative treatment in order to minimize symptoms arising from the underlying pathology, which may occur in cases of metastatic prostate cancer (mPCa) [4].

The primary treatment has been accepted and recommended in other metastatic malignancies with good morbidity and mortality outcomes [5, 6].

Some emerging theories point to the accomplishment of primary therapy, although still without strong scientific evidence.

### 3.2. Control of Local Disease

Advanced stages of prostate cancer increase patients' morbidity and mortality risk by local progression and/or establishment of metastasis. Local progression may cause invasion of adjacent structures leading to pain, urinary retention due to invasion of the bladder and/or urethra, obstruction of the rectum, establishment of rectourethral or rectovesical fistulas, and infiltration of the pelvic nerve bundles. These factors impair the patients' quality of life and may impact survival. In some cases, it is necessary to perform palliative treatment. The frequency of these complications may be related to whether or not local therapy was performed in a phase of localized disease [7, 8].

It is scientifically plausible that uncontrolled local disease is the source of circulating tumor cells that can establish distant metastases and even recolonize the primary tumor itself with more aggressive and hormone-resistant cell clones [5, 9]. It is also assumed that the primary lesion may produce factors responsible for maintenance of circulating cancer cells' viability [10].

Combination of androgen deprivation therapy (ADT) with a primary treatment (radical prostatectomy or radiotherapy) may be more efficient in terms of local progression control than just isolated ADT. By controlling the local disease through directed local therapy, local symptoms conditioned by the growth and progression of the primary lesion are prevented or delayed [5, 11, 12].

### 3.3. Metastasis Establishment Prevention/Control

The primary carcinoma, host cells, and metastases are part of a communication circuit connected by molecular pathways, which is responsible for microenvironment changes in certain regions, premetastatic niches, which are responsible for subsequent metastatic pattern. Metastatic establishment seems to occur in pulses [5, 19].

After primary cancer's surgical ablation, circulating tumor cells are unable to establish new metastases. It is biologically plausible that the uncontrolled primary lesion promotes the growth and appearance of new tumoral lesions. However, this capacity is not a unique capacity of the primary lesion, since some metastases may establish and promote the growth of new metastases too. Antwi and Everson also demonstrated that the probability of death from any cause increases with the number of established metastases [4, 9, 20–22].

The abscopal effect, a phenomenon that corresponds to the regression of distant metastases during primary treatment, is another explanation for a potential benefit of local treatment. Radiotherapy promotes antitumor immune reactions directed to the primary lesion and metastases by improving the cross-presentation of antigens by activating cytotoxic CD8+ T cells. Thus, radiotherapy when combined with ADT has an effect on the tumor's genetic expression, potentiating apoptosis [12].

It is logical to think that directed local therapy of primary carcinoma reduces the likelihood of metastasis onset, establishment, and growth through the breakdown of the communication between the different cells involved in the process, not allowing the creation of the necessary environment for implantation of circulating tumor cells in distant sites and preventing signaling that promotes their growth.

### 3.4. Improved Hormonotherapy and Chemotherapy Efficacy

As stated before, primary treatment with radiotherapy potentiates the effect of hormonal manipulations due to the abscopal effect [12]. The progression of the primary tumor leads to increased genetic and phenotypic heterogeneity, which will lead to appearance of cell colonies with different characteristics: increased proliferative capacity, metastatic potential, and resistance to treatments [7]. Some colonies resistant to castration will be further selected by ADT, which will promote their growth, conditioning the development of resistance to castration, a capacity that many of these tumors show after a few months of conventional therapy [6].

A recent study has shown that primary tumor therapy, whether radical prostatectomy (RP) or radiotherapy (RT), increases the time to the development of castrate-resistant prostate cancer (8 years compared to 4 years in the control group) [23].

Thus, by eliminating the possibility of increased heterogeneity, we may increase the likelihood of long-lasting response to systemic therapies associated with primary treatments, since evidence suggests that the prostate is a focus of resistance to currently recommended regimens, which contributes to accelerating progression [7, 12, 24].

### 3.5. Results of Primary Therapy Performed in Other Metastatic Cancers

Several studies show that primary tumor therapy is associated with increased survival and better response to systemic treatments in patients diagnosed with metastatic cancer disease, including renal cell carcinoma, colon cancer, breast cancer, ovarian cancer, and glioblastoma. Studies suggest that prostate cancer may have similar behavior [10, 25].

### 3.6. Radical Prostatectomy and Radiotherapy as Primary Treatment of mPCa

Recent data suggests that performing RP or RT in patients duly selected with mPCa is associated with better oncological outcomes and better overall survival (OS), cancer-specific survival (CSS), and prostate cancer-free survival (PCFS) [4, 25]. Patient survival increases regardless of M-stage according to American Joint Committee on Cancer (AJCC) [13].

Antwi and Everson conducted a retrospective study using Surveillance, Epidemiology, and End Results Program (SEER) data. Average survival time after diagnosis was 29 months for patients in the RP group, 31 months in brachytherapy (BT) group, and 17 months in no local treatment (NLT) group. It was concluded that patients receiving local therapy had higher survival rates and a lower probability of dying from mPCa (15.3% in the PR group, 28.3% in the BT group, and 45.4% in the NLT group) [4] (Table 1).

Culp et al. through the SEER database compared the results of performing local therapy (RP or BT) versus patients without local treatment. Overall survival at 5 years was 67.4% in the RP group, 52.6% in the BT group, and 22.5% in the NTL group. In the case of specific survival related to PCa, patients in the RP and BT groups showed better results than the NLT group (75.8% and 61.3% compared to 48.7% of the latter group). OS and disease-specific survival (DSS) were higher in patients <70 years, but only OS was significantly higher in patients ≥70 years with local treatment compared to the NLT group. In the prostate-specific antigen (PSA) based groups, OS and DSS were higher in local treatment (LT) group patients with PSA < 20. In the PSA ≥ 20 group, the probability of OS was significantly higher. Considering the extent of metastases, men who underwent RP had decrease in cancer-specific mortality (CSM) not influenced by M-stage and higher OS in groups M1b and M1c. In the case of patients receiving BT, the results were overlapping [13] (Tables 2–5).

Both SEER-based studies excluded external RT from the analysis, since it might be difficult to tell which patients received it with a definitive or palliative intention [13].

Löppenberg et al. in a National Cancer Database (NCDB) based study assessed the impact of local therapy on men with PCa at diagnosis. Overall mortality-free survival (OM-FS) rate was 50% in the total population studied, 63% in LT group, and 48% in NLT group. In the LT group associated with ADT within 6 months of diagnosis, the probability of 3-year survival was 57% and it was 69% in LT group, while in the LT + ADT group it was 48%. When separated by treatment, survival at 3 years was higher in the BT patients (80%) followed by the RP patients (78%) and finally RT patients (60%) [14] (Table 6).

Satkunasivam et al. analyzed data from SEER collected between 2004 and 2009 of men with mPCa, taking into account their treatment modality. OS rate at 3 years was 73% in the RP group, 72% in the intensity-modulated radiotherapy (IMRT), 37% in the conformal radiation therapy (CRT), and 34% in the NTL. DSS at 3 years was 79% in RP group, 82% in IMRT group, 49% in CRT group, and 46% in NLT group [15] (Table 7).

Gratzke et al. followed the same direction of previously published studies using data from the RCM collected between 1998 and 2010. Survival probability at 5 years was superior in the RP group compared to NLT group (55% versus 21%) [26].

Cho et al. carried out a cohort study with the purpose of analyzing the efficacy and safety of RT treatment in mPCa patients. The established indications for RT included metastatic lesions conditioning pain, fracture risk, and neurological complications. Virtually all patients (96%) started ADT at diagnosis. RT group had a 69% OS at 3 years, which was significantly higher than the 43% of the NLT group. Biochemical failure-free survival (BCFFS) value at 3 years was also higher in the RT group when compared to NLT group (52% and 16%, resp.) [16] (Table 8).

Rusthoven et al. looked through the data recorded between 2004 and 2012 in NCDB to evaluate the outcome of RT therapy in patients with mPCa at diagnosis. ADT combined with prostate-directed RT had longer OS (53 months) versus 29 months of isolated ADT therapy. Patients who received ADT + RT had an OS probability of 62% at 3 years, 49% at 5 years, and 33% at 8 years compared to 43%, 25%, and 13%, respectively, for those who received ADT only [17] (Table 9).

Heidenreich et al. developed the first case-control study addressing this subject and although with a small population, the criteria used to select individuals allowed to reach important conclusions. Group 1 included 23 patients with mPCa with low volume bone metastases who underwent cytoreductive radical prostatectomy (CRP), since 6 defined criteria were satisfied: completely resectable PCa assessed by transrectal ultrasonography and rectal examination, three or less bone metastases, absence of retroperitoneal lymphatic metastases, absence of pelvic lymphadenopathy greater than 3 centimeters, absence of visceral metastases, and signed informed consent. This group has been given luteinizing hormone-releasing hormone (LHRH) analogues for at least 6 months in combination with bicalutamide. A second control group (group 2) consisted of 38 men treated with ADT, without any local therapy until there was progression and were submitted to intervention only if local symptoms were present. Comparing the two groups according to OS rate and CSS rate, group 1 had 91,3% OS rate and group 2 78,9%. CSS rate was 95,6% in group 1 and 84,2% in group 2 [18] (Table 10).

### 3.7. Complications of Local Treatment in mPCa

Primary treatment modalities that may be offered in cases of PCa also entail complications. However, current evidence shows that these are not worse than those usually seen in these procedures for primary localized disease.

Considering the postoperative complications over a 90-day period, according to a 2015 study, 79.2% of patients did not experience any complication. Of the 106 men, only 21 (19.8%) experienced complications, 9 (8.5%) had lymphocele, 7 (6.6%) had anastomotic leakage, and 5 (4.7%) had a surgical scar infection. Six (27.3%) of the patients had 2 complications. M1a and M1b (19.4% versus 21.4% of patients) did not differ significantly. Postsurgical urinary continence at 90 days revealed that 38 (64.4%) of the 59 patients reported were reestablished, and only 11 (18.6%) of the 59 patients had moderate-to-severe incontinence. Based on these data, RP in cases of mPCa is safe and reliable and does not lead to more complications or increased mortality compared to nonmetastatic patients [27].

In Heidenreich et al.'s study, the mean time of hospitalization was 7.8 days with an average of 5.6 days of bladder catheterization. Intraoperatively, the blood loss had an average value of 335 ml, with preoperative and postoperative hemoglobin values of 13.1 mg/dl and 11.8 mg/dl, respectively. Three patients (13%) who developed lymphocele were found, of which 2 (8.7%) were resolved by percutaneous drainage and 1 (4.3%) by laparoscopic marsupialization. Two patients (8.7%) developed deep vein thrombosis (DVT) and one of them had pulmonary embolism. Analyzing the data on urinary continence, 91.3% presented urinary continence with 1 or fewer diapers per day [18].

Cho et al. evaluated the toxicity of RT follow-up and weekly hematologic evaluation. None of the 38 patients who performed RT directed to the primary lesion had severe effects due to this therapeutic modality. However, 4 men (11%) had grade 3 thrombocytopenia and 3 (8%) had grade 3 leukocytopenia [16].

### 3.8. Identify Candidates for Local Treatment of mPCa at Diagnosis

First of all, the primary tumor should have characteristics that allow local treatment to be performed. When opting for a surgical modality, the primary neoplastic lesion should be resected with appropriate safety margins. If the lesion is too extensive, neoadjuvant ADT followed by a local therapeutic modality chosen (radical prostatectomy or targeted radiotherapy) may be considered [7].

Independent factors that have a negative influence are age above 70 years, cT4 stage or high-grade disease, PSA ≥ 20 ng/ml, and pelvic lymphadenopathies. The presence of 3 or more of these factors conditions CSS and OS is the same between patients who performed local treatment and those who did not [13]. Metastasis degree, location, and number can also influence outcome. Meanwhile, patients with visceral metastases have worse results [16, 28].

## 4. Conclusion

In recent years, there has been growing interest with the possibility of offering treatments directed to the primary tumor in cases of metastatic prostate carcinoma at diagnosis. It is thus sought to achieve local disease control and consequent control of systemic disease by performing radical prostatectomy or targeted radiation therapy. There are several rational-based explanations and strong evidences that support this new stream of thought.

Several studies have suggested the advantages in patient overall survival and cancer-specific survival when compared to current therapeutic approaches. In virtually all studies, radical prostatectomy has a slight advantage over directed radiotherapy. However, caution should be exercised when analyzing existing data because we based our review on retrospective studies that scrutinized information from databases in which it is not always possible to obtain all relevant information about all patients. The authors themselves generally point out this gap in their studies, which does not allow conclusions to be drawn with high scientific evidence. Selection bias and previous or concomitant treatments are the general limitations of the studies databases.

At this point, one of the biggest questions is to realize which patients may actually benefit from local radical therapies. The studies analyzed in general indicate factors that condition less favorable prognoses that may in the future be the basis for the selection criteria.

On the other hand, recently published data for the treatment of mPCa (CHAARTED, STAMPEDE, and LATITUDE trials) bring some more confounding variables and systemic medication might be more beneficial than local treatments in the metastatic setting.

By now, it is impossible to say with certainty that there is an obvious advantage in performing primary treatment in metastatic prostate carcinoma. However, it is possible to affirm that the evidence begins to point in this direction and that we may be close to a paradigm shift. Prospective multicentric studies are indubitably needed in this field at the same time that new standard therapies (docetaxel or abiraterone plus hormonotherapy) will make recruitment for local treatments more difficult.

## Figures and Tables

**Table 1:** Mean survival time and disease-specific mortality by therapeutic modality, adapted from Antwi and Everson (2014) [4].

Therapeutic modality	Average survival time after diagnosis (months)	DSM (%)
RP	29	15,3
BT	31	28,3
NLT	17	45,4

DSM: disease-specific mortality; RP: radical prostatectomy; BT: brachytherapy; NLT: no local treatment.

**Table 2:** Overall survival and cancer-specific survival by therapeutic modality, adapted from Culp et al. (2014) [13].

Therapeutic modality	5-year OS rate (%)	5-year CSS rate (%)
RP	67,4	75,8
BT	52,6	61,3
NLT	22,5	48,7

OS: overall survival; CSS: cancer-specific survival; RP: radical prostatectomy; BT: brachytherapy; NLT: no local treatment.

**Table 3:** Overall survival and disease-specific survival by age and therapeutic modality, adapted from Culp et al. (2014) [13].

Age/therapeutic modality	5-year OS rate (%)	DSS rate (%)		
		1 year	3 years	5 years
<70 years				
NLT	28,9	86,1	57,7	45,8
RP	71,2	96,7	86,9	82,0
BT	57,4	92,2	73,9	65,2

≥70 years				
NLT	18,1	80,1	58,6	49,5
RP	50,3	86,7	70,8	63,5
BT	48,5	86,2	69,9	62,5

OS: overall survival; DSS: disease-specific survival; RP: radical prostatectomy; BT: brachytherapy; NLT: no local treatment.

**Table 4:** Overall survival and disease-specific survival by PSA and therapeutic modality, adapted from Culp et al. (2014) [13].

PSA/therapeutic modality	5-year OS rate (%)	DSS rate (%)		
		1 year	3 years	5 years
<20 ng/ml				
NLT	33,7	87,3	66,6	57,9
RP	77,1	96,5	89,9	86,7
BT	71,2	95,3	86,5	82,3

≥20 ng/ml				
NLT	19,8	81,4	55,6	44,8
RP	55,7	88,8	71,3	63,0
BT	37,3	82,9	58,5	48,1

PSA: prostate-specific antigen; OS: overall survival; DSS: disease-specific survival; RP: radical prostatectomy; BT: brachytherapy; NLT: no local treatment.

**Table 5:** Overall survival and disease-specific survival by stage M according to the AJCC criteria and therapeutic modality, adapted from Culp et al. (2014) [13].

AJCC M-stage/therapeutic modality	5-year OS rate (%)	DSS rate (%)		
		1 year	3 years	5 years
M1a				
NLT	35,1	93,4	73,3	61,4
RP	64,3	98,4	92,9	89,1
BT	54,7	96,3	84,1	76,2

M1b				
NLT	22,9	84,1	59,6	48,4
RP	70,1	94,1	83,4	77,6
BT	55,0	89,2	71,0	61,9

M1c				
NLT	18,6	75,6	50,4	43,0
RP	60,7	91,1	80,0	75,6
BT	53,4	85,4	68,3	62,1

AJCC: American Joint Committee on Cancer; OS: overall survival; DSS: disease-specific survival; RP: radical prostatectomy; BT: brachytherapy; NLT: no local treatment.

**Table 6:** Overall mortality-free survival and overall survival by therapeutic modality, adapted from Löppenberg et al. (2016) [14].

Therapeutic modality	3-year OM-FS rate (%)	3-year OS rate (%)
TP	50,0	
NLT	48,0	
NLT + ADT		48,0

LT	63,0	69,0
LT + ADT		57,0
RP		78,0
BT		80,0
RT		60,0

OM-FS: overall mortality-free survival; OS: overall survival; TP: total population; NLT: no local treatment; ADT: androgen deprivation therapy; RP: radical prostatectomy; BT: brachytherapy; RT: radiotherapy.

**Table 7:** Overall survival and disease-specific survival by therapeutic modality, adapted from Satkunasivam et al. (2015) [15].

Therapeutic modality	3-year OS rate (%)	3-year DSS rate (%)
NLT	34	46,0
CRT	37	49,0
IMRT	72,0	82,0
RP	73,0	79,0

OS: overall survival; DSS: disease-specific survival; NLT: no local treatment; CRT: conformal radiation therapy; IMRT: intensity-modulated radiotherapy; RP: radical prostatectomy.

**Table 8:** Overall survival and biochemical failure free survival by therapeutic modality, adapted from Cho et al. (2016) [16].

Therapeutic modality	3-year OS rate (%)	BCFFS (%)
TP	48,2	25,0
RT	69,0	52,0
NLT	43,0	16,0
Palliative RT	50,0	10,0
Without palliative RT	40,0	20,0

OS: overall survival; BCFFS: biochemical failure-free survival; TP: total population; RT: radiotherapy; NLT: no local treatment.

**Table 9:** Overall survival and overall survival rates by therapeutic modality, adapted from Rusthoven et al. (2016) [17].

	OS (months)	3-year OS rate (%)	5-year OS rate (%)	8-year OS rate (%)
RT + ADT	53	62,0	49,0	33,0
ADT	29	43,0	25,0	13,0

OS: overall survival; RT: radiotherapy; ADT: androgen deprivation therapy.

**Table 10:** Overall survival and surgery-free survival rate by therapeutic modality, adapted from Heidenreich et al. [18].

Therapeutic modality	OS rate (%)	CSS rate (%)	Surgery-free survival rate
RP	91,3	95,6	100,0
NLT	78,9	84,2	71,1

OS: overall survival; CSS: cancer-specific survival; RP: radical prostatectomy; NLT: no local treatment.
